# Smartphone-based platforms implementing microfluidic detection with image-based artificial intelligence

**DOI:** 10.1038/s41467-023-36017-x

**Published:** 2023-03-11

**Authors:** Bangfeng Wang, Yiwei Li, Mengfan Zhou, Yulong Han, Mingyu Zhang, Zhaolong Gao, Zetai Liu, Peng Chen, Wei Du, Xingcai Zhang, Xiaojun Feng, Bi-Feng Liu

**Affiliations:** 1 The Key Laboratory for Biomedical Photonics of MOE at Wuhan National Laboratory for Optoelectronics - Hubei Bioinformatics & Molecular Imaging Key Laboratory, Systems Biology Theme, Department of Biomedical Engineering, College of Life Science and Technology, Huazhong University of Science and Technology, Wuhan, 430074 China; 2 School of Engineering and Applied Sciences, Harvard University, Cambridge, MA 02138 USA

**Keywords:** Computational biology and bioinformatics, Laboratory techniques and procedures, Materials for devices, Biomedical engineering, Microfluidics

## Abstract

The frequent outbreak of global infectious diseases has prompted the development of rapid and effective diagnostic tools for the early screening of potential patients in point-of-care testing scenarios. With advances in mobile computing power and microfluidic technology, the smartphone-based mobile health platform has drawn significant attention from researchers developing point-of-care testing devices that integrate microfluidic optical detection with artificial intelligence analysis. In this article, we summarize recent progress in these mobile health platforms, including the aspects of microfluidic chips, imaging modalities, supporting components, and the development of software algorithms. We document the application of mobile health platforms in terms of the detection objects, including molecules, viruses, cells, and parasites. Finally, we discuss the prospects for future development of mobile health platforms.

## Abstract (web summary)

Smartphone-based mobile health platforms have drawn increasing attention from researchers developing point-of-care testing devices. Here the authors summarize recent progress and future directions of approaches combining microfluidics and artificial intelligence.

## Introduction

Disease diagnosis in resource-limited environments remains a significant challenge, since most existing biosensing technologies rely on advanced infrastructure and well-trained personnel, which limits their applications in point-of-care testing (POCT) scenarios. Ideally, a POCT platform should have characteristics including ease of operation, short analysis time, low price, high sensitivity, and specificity to meet the requirements of health monitoring in a POCT setting^1–9^.

With the advances in optics technology, materials and software engineering, electrical microchip technology and so on, smartphones are becoming smaller and more powerful with an enhanced user experience, providing a promising platform for POCT developments^10–16^. In recent years, microfluidic accessories have emerged to explore the potential of smartphones for POCT, especially the microfluidic detection modules based on smartphone complementary metal oxide semiconductor (CMOS) cameras, which are also known as the mobile health (mHealth) platform^17,18^. The World Health Organization defines mHealth as the medical and public health practice supported by mobile devices. Mobile devices include smartphones and tablets, as well as devices and wearables that provide real-time patient monitoring. In this article, we mainly focus on the advances in smartphone-based mHealth platforms that implement microfluidic detection and image-based analysis.

The mHealth platforms take advantage of microfluidic technology such as automation, integration, miniaturization and multi-functions, which is ideal for health monitoring in a POCT setting. As the computing power of digital devices such as computers and smartphones significantly increases, it facilitates the development of artificial intelligence algorithms that require powerful computing and large amounts of data. Recently, deep learning algorithms such as the convolutional neural network (CNN) algorithm have been widely applied to image processing in mHealth^19^. The combination of microfluidic accessories and artificial intelligence algorithms has inspired researchers worldwide to come up with new POCT tools^20–32^. On the one hand, mHealth takes advantage of the microfluidic approach to measure traditional biomarkers more accurately, which are detected by smartphone CMOS cameras and analyzed with machine learning algorithms. On the other hand, unique patterns and features extracted by machine learning algorithms from the data can reflect biomedical information that is difficult to obtain by traditional methods^33–35^.

**Fig. 1:**
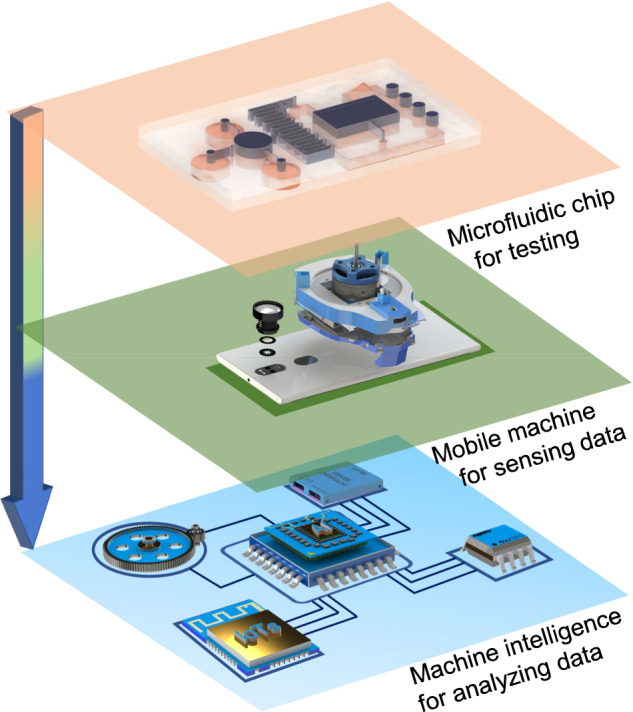
Architecture of machine learning-enabled mHealth platforms for POCT. The microfluidic chip is responsible for biosample processing and testing. The resulting signal is acquired by the mobile machine and preprocessed by software installed on the smartphone. After data is transmitted to the cloud server, it can be stored and further analyzed by machine intelligence. IoTs and AI denote internet of things and artificial intelligence, respectively.

At present, an ideal mHealth platform includes three parts (Fig. 1), that is, a microfluidic chip, a mobile machine, and machine intelligence. The microfluidic chip is responsible for biosample processing and testing. The resulting signal is sensed by the mobile machine and preprocessed by software installed on the smartphone. After data is transmitted to the cloud server, it can be stored and further analyzed by corresponding machine intelligence.

In this work, we reviewed the progress of research on mHealth platforms in recent years, especially that regarding microfluidic chips for testing and mobile machines for sensing data, including imaging modalities and supporting components.

We will further discuss machine intelligence for analyzing data in mHealth, particularly the rapid development of deep learning algorithms. Current applications of mHealth will also be documented and future development will be discussed.

## Microfluidic chips for testing

It is essential to use microfluidic technology to realize the integration and miniaturization of complex biological analysis on mHealth platforms, especially those for applications in remote areas. In comparison to the microfluidic chips commonly used in laboratories, those used on mHealth platforms often have more limitations because many large devices such as pumps and heaters cannot be directly used on them. Therefore, in many cases, microfluidic chips are employed as simple carriers containing blood^36^, tissue^37^, parasites^38^, and other biological samples.

Besides being used as a slide, microfluidic chips could be designed with simple reaction chambers for relatively complex detection. Kanakasabapathy et al. developed a mHealth platform that could detect the number of CD4 cells to diagnose Acquired Immune Deficiency Syndrome (AIDS)^39^. CD4 antibody was immobilized in a reaction chamber. After CD4 cells were captured in the chamber, a photo was acquired through the mobile phone camera, and an application program was then activated to count the numbers of captured CD4 cells for AIDS diagnosis. The design of microfluidic chips with reaction chambers is simple and effective for many applications on mHealth platforms. However, it is difficult to realize automated detection on such microfluidic chips because the detection process largely depends on manual operation by the user, which greatly reduces the practicability of mHealth platforms.

**Fig. 2:**
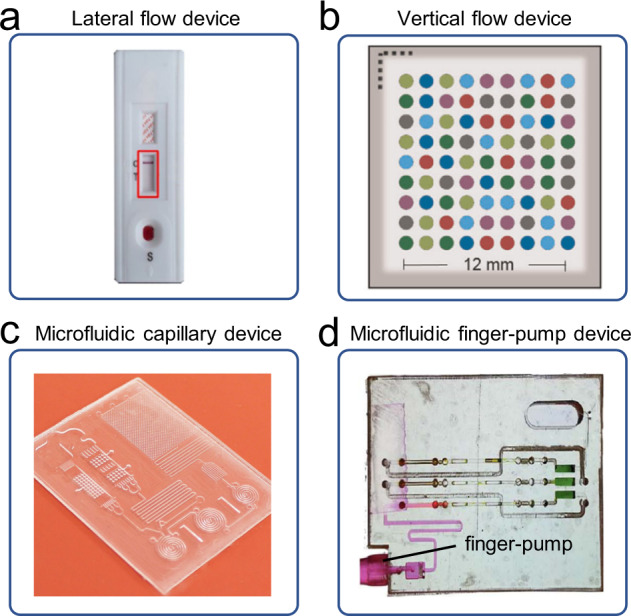
Microfluidic chips for testing. **a** A lateral flow device (figure adapted with permission from Turbé et al. ^7^). **b** A vertical flow device (figure adapted with permission from Ballard et al. ^19^). **c** A microfluidic capillary device (figure adapted with permission from Ghosh et al. ^47^). **d** A microfluidic finger-pump device (figure adapted with permission from Comina et al. ^48^).

The lateral flow device is also a cost-effective detection method with a relatively low requirement for external equipment. The simple and understandable signal amplification and display mode allow users to take photos with mobile phones for identification and analysis without microscopic imaging. For instance, Turbé et al. demonstrated the use of deep learning to classify images of rapid Human Immunodeficiency Virus (HIV) tests, which were collected in rural South Africa by fieldworkers using newly developed image capture protocols with the Samsung SM-P585 tablet^7^. Deep learning algorithms were trained to classify tests as positive or negative from a library of 11,374 images. A pilot field study of the algorithms deployed as a mobile application demonstrated high levels of sensitivity (97.8%) and specificity (100%) compared with traditional visual interpretation by humans and reduced the number of false positives and negatives (Fig. 2a).

The capillary force can drive the fluid without external equipment. However, the uniform flow rate of the lateral flow device requires several micrometer pore sizes of the paper, which limit the biomolecule capturing capability and assay sensitivity. Moreover, it is difficult for lateral flow devices to realize multiplexed detection^40^. One of the ways to improve the lateral flow device is to flow samples vertically rather than in parallel, known as a vertical flow assay (VFA). A typical vertical flow device contains a porous membrane with separated spots for multiplexing assays simultaneously^19^ (Fig. 2b), and the detection results can be readout by the naked eye or a smartphone reader, which holds high potential for mHealth applications.

It is better to employ microfluidic chips designed with microchannels for more complex applications. Traditional micropumps are usually too large to be integrated into mHealth platforms for fluid control. To overcome this issue, researchers have developed innovative micropumps, such as capillary, finger, and passive vacuum pumps^41–46^, for driving fluid on mHealth platforms. Ghosh et al. demonstrated a microfluidic chip containing a capillary pump for microchannel capillary flow assay (MCFA) on a mHealth platform^47^. The immunoassay on the chip was entirely controlled by channel geometry and surface properties without any external pumping (Fig. 2c). Comina et al. presented an autonomous disposable system integrating a finger pump supporting repeated pumping actions^48^, a calibration range, and 3D-printed optics for universal coupling to cellphone cameras (Fig. 2d). By integrating heater and other process control units, it is possible to realize applications such as nucleic acid detection on mHealth Platforms^49–53^. For instance, Gou et al. developed a low-cost, portable, and robust mHealth platform for highly accurate DNA quantitative analysis^54^, integrating thermal cycling control, on-chip digital polymerase chain reaction, data acquisition, and result analysis.

Microfluidic chips have been the dominant choice for sample processing on mHealth platforms, which are also increasingly adapted to artificial intelligence algorithms. POCT detection on mHealth platforms in the early stage is to obtain intuitive detection results so that inspectors can directly identify them. Therefore, in many early works, even if the artificial intelligence algorithm is used, it is only simple transplantation of image recognition, and there is no microfluidic chip specially designed for artificial intelligence algorithm. In recent years, more and more microfluidic chip designs have emerged for analysis by artificial intelligence algorithms.

## Mobile machines for sensing data

One of the essential factors in the mHealth platform is the hardware structure, ensuring the quality of the obtained images or data. A superior hardware structure can not only reduce the cost, but also simplify the operation process, which is beneficial in resource-limited areas where medical diagnosis resources are scarce. A complete mHealth platform based on microscope cameras should contain three basic hardware structures, including mobile phones, imaging modalities and supporting components.

As the core of the mHealth platform, the smartphone is the key to the hardware structure. With the development of integrated circuit (IC) design technology, the resolution of embedded CMOS image sensors has greatly increased, allowing smartphones to detect biomarkers on a relatively small scale with CMOS cameras^55–57^. In addition, the computing capabilities of embedded central processing units (CPUs) in smartphones have been enhanced rapidly. It is possible for smartphones to process and analyze biomedical images in real time. Current mHealth platforms are mostly designed according to various parameters of mobile phones to take advantage of the mobile phone system for POCT applications. For example, embedded adapters need to be adjusted according to the size of the mobile phone. The external lens must be designed according to the original focal length of the mobile phone to achieve a good, clear amplification. Differences in CMOS components in mobile phones will lead to differences in acquired images, which must be processed by the corresponding algorithms before adaptation. The computing performance of the mobile phone must meet the requirements of algorithms with a large amount of computation.

To detect micro-scale objects with the smartphone camera, it is necessary to develop accessories for optical magnification, including 3D-printed phone adapters, lenses, batteries, light sources, controllers, and motors. In the following sections, hardware developments related to mHealth platforms will be introduced in detail based on two aspects: imaging modalities and supporting components. It is expected to provide the readers with a guide for designing the mHealth platform that is suitable for their own needs.

### Imaging modalities

Currently, the imaging modalities for mHealth platforms can be classified into two major categories, which are bright field and fluorescence imaging. Bright field imaging can be subdivided into lens-free and lensed imaging. The microscopic image quality obtained by different imaging modalities varies greatly, including resolution, field of view (FOV), imaging depth, and signal-to-noise ratio (SNR). Thus, these imaging modalities have their own advantages in detecting objects of different scales, and an appropriate imaging modality must be chosen according to the detection objects. Once an imaging modality is determined, the corresponding hardware structure, especially the optical structure, can be designed accordingly, as well as a suitable software algorithm for analysis of the resulting imaging data. Supplementary Table 1 summarizes the advantages and disadvantages of current imaging modalities discussed in the rest of this section.

In bright field imaging, since the illumination light enters the objective lens after passing through the sample, the resulting image contrast is relatively high, giving a clear morphological image of the sample. It is usually used to observe objects with a clear structure on a micron scale, such as cells, tissues and parasites.

**Fig. 3:**
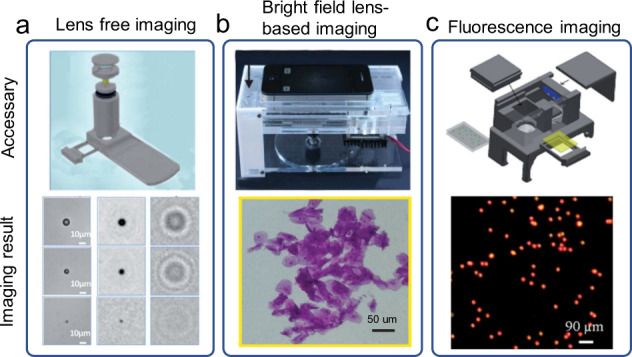
Imaging modalities. **a** Schematic diagram of an optical accessary for lens-free imaging and its imaging result (figure adapted with permission from Tseng et al. ^58^). **b** Picture of an optical accessary for Bright field lens-based imaging and its imaging result (figure adapted with permission from Switz et al. ^62^). **c** Schematic diagram of an optical accessary for fluorescence imaging and its imaging result (figure adapted with permission from Zhu et al. ^197^).

As one of the bright field imaging modalities, lens-free imaging has been applied to the mHealth platform for its simple and compact optical structure. Upon removal of the original lens from the mobile phone, it directly uses the embedded CMOS image sensor for data collection^58–60^. As a result, lens-free imaging system can provide a relatively larger FOV than a lensed imaging system with higher image resolution. For example, a mHealth platform was previously demonstrated by Tseng et al.^58^ which employed holographic image reconstruction to capture the amplitude and phase information of the objects with the embedded CMOS sensor in a smartphone (Fig. 3a). When the spatial and temporal coherence of the light source is good enough, the fringes collected by the camera can be regarded as interference fringes. In this work, the authors did not use expensive laser components to generate coherent light sources. Instead, an LED was used to emit incoherent light, which passed through an aperture with a diameter of about 100 µM to generate spatial coherence. These resulting interference fringes could then be used to obtain the intensity and phase information of the object in the focusing plane to reconstruct a holographic image with a relatively high resolution through the diffraction image reconstruction algorithm. To further miniaturize the size of the lens-free imaging system, Lee and Yang reported the use of ambient illumination in the following work^59^, which enabled shadow imaging without the need for LEDs or batteries. Shadow imaging was relatively simple. It only required the projection of the object on the CMOS sensor without the need for high coherence of the light source. However, the resulting image resolution was relatively low due to diffraction issues, which could be improved by manual angle scanning and the use of a super-resolution reconstruction algorithm.

Although a lens-free imaging system is simple and compact, it takes a long time and a lot of computing resources to generate high-resolution images with super-resolution reconstruction algorithms. In addition, it involves the removal of the smartphone camera lens, which may damage the integrity of the smartphone. Thus, it is likely to reduce user experience for applications. In contrast, there is no such issue for lensed imaging systems. The optical magnification (*M*) of a lensed imaging system can be calculated as *M* = $\frac{{f}_{1}}{{f}_{2}}$, where ${f}_{1}$ is the focal length of the built-in lens of the smartphone camera and ${f}_{2}$ is the focal length of the external lens. The resolution of lensed imaging is inversely proportional to its FOV. Therefore, the use of an external lens of a shorter focal length can result in higher magnification and better spatial resolution, but correspondingly, the FOV will be smaller. Since the observation of particles of different specifications requires different resolutions and FOV, it is necessary to make a trade-off between the resolution and FOV. For example, Smith et al. described a mHealth platform that used a single ball lens for magnification in place of a microscopic objective^61^. It is cost-effective for constructing the detection system, but images were obtained with significant aberrations that degraded the image quality over the bulk of the FOV. To capture high-quality wide-field microscopic images, Switz et al. demonstrated that a reversed mobile phone camera lens could be used together^62^ with an intact mobile phone camera to overcome the limitations of the previous design (Fig. 3b). In addition to the above-mentioned methods, it is also possible to use deep learning image enhancement methods to improve image resolution and increase FOV with field-by-field scanning. However, these methods are trading time for space, which elongates the time for processing to enhance image resolution or FOV.

In fluorescence imaging, the light of a specific wavelength irradiates the sample, and the emitted fluorescence is collected through the objective. As long as relevant imaging parts such as light sources and filters can be installed onto the mobile phone as accessories, fluorescence imaging can also be conducted on mHealth platforms. Compared to bright field imaging, fluorescence imaging has higher sensitivity and specificity, and it is more suitable for detecting micro- and nano-scale biological targets^63^. The lens for fluorescent imaging does not require high resolution but a relatively large FOV (Fig. 3c). It is conducive to employing fluorescence imaging on mHealth platforms. In addition, the algorithms for processing fluorescence images are less demanding than those for bright field imaging. Quantitative analysis can also be achieved by merely using counting algorithms.

However, fluorescence imaging often yields images of low contrast and it is difficult to observe the shape of the detection objects. The quality of the obtained fluorescence images is related to the SNR. To enhance the SNR, it is important to select an appropriate light source and filter for fluorescence imaging. Since traditional laser light sources are expensive and cumbersome, low-cost filters and LEDs have been the major choice for fluorescence imaging on mHealth platforms. The resulting images usually have strong background lights, and the signal-to-noise ratio of the images is relatively low. Although image quality can be further improved by denoising algorithms such as machine learning algorithms, it is still inferior to those obtained by bright field imaging. Besides limited image quality, fluorescence imaging often requires sample preprocessing, and such operations might not be friendly to untrained users.

### Supporting components

After the imaging modality is determined, the supporting component of the mHealth platform can be established. The physical components of a mHealth platform typically include an optical imaging part, process control unit, structural part, motor, heater, finger pump and so on.

The optical imaging part is the basic component of a mHealth platform, which is dedicated to generating the imaging data. The microscopic lens is the core component of the optical imaging part with specifications such as numerical aperture, magnification, imaging resolution, FOV, and marginal effect. Different specifications may be employed according to the scenario of applications, but typically, LEDs and batteries are necessary for providing light sources. Filters are required in fluorescence imaging to provide excitation light.

**Fig. 4:**
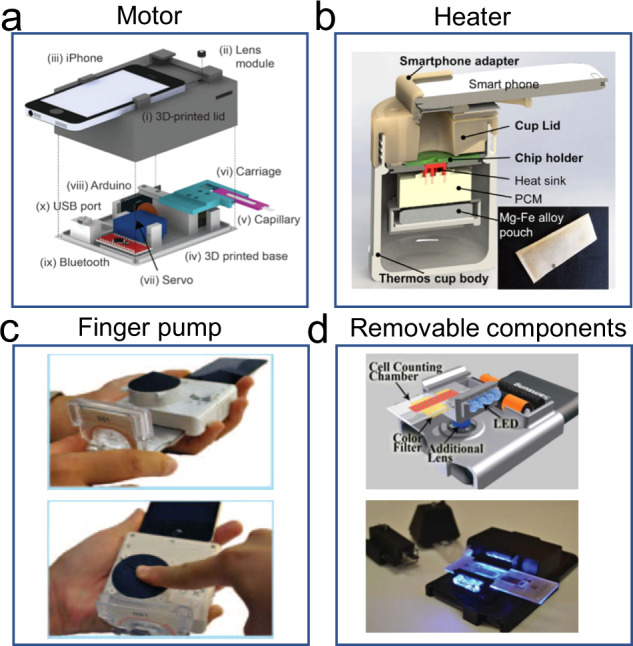
Supporting components. **a** Motor for FOV scanning. FOV denotes field of view (figure adapted with permission from D’Ambrosio et al. ^18^). **b** Heater for temperature control. PCM denotes phase-change material (figure adapted with permission from Liao et al. ^51^). **c** Finger pump for flow driving (figure adapted with permission from Laksanasopin et al. ^17^). **d** Attachment with a port for interchangeable components interchanging (figure adapted with permission from Zhu et al. ^70^).

Process control units such as the controller, motor, heater and micropump are usually selected according to the imaging modality and microfluidic chip operations. The controller is the kernel of the process control unit, which generally uses a Micro Control Unit (MCU) or embedded system to automatically control the whole process of various integrated components, allowing operations such as USB and Bluetooth communication, motor motion, heating, and flow control. Motors are often employed to achieve FOV scanning with high resolution since the resolution of the microscopic lens is inversely proportional to its FOV. For instance, D’Ambrosio et al. reported on a mHealth testing instrument using a dynamic video method^18^, which could achieve FOV scanning of blood parasites with a motor-based automated stage (Fig. 4a). It could automatically record a video of the test sample and calculate a differential image by averaging, subtracting, and morphologically filtering each frame of the video to obtain a count of blood parasites. For mHealth systems involving nucleic acid amplification, such as polymerase chain reaction (PCR) and loop-mediated isothermal amplification (LAMP), heaters must be used for accurate temperature control. Mauk et al. previously described nucleic acid-based amplification tests (NAATs) mHealth platform for quantifying HIV viral load in blood samples^64^, which used a heater to accurately control the reaction temperature (Fig. 4b). However, since the batteries that powered the heater were too heavy to carry, some researchers instead developed solar and chemical thermal heating methods. A similar issue also occurred for the use of micropumps in mHealth systems. The volume and energy consumption of micropumps are usually too large to be adapted to the mHealth platform^65–69^. To overcome this issue, Laksanasopin et al. eliminated the use of a power-consuming electrical pump by employing a one-push vacuum^17^, where a user mechanically activates a negative pressure chamber to move a sequence of reagents prestored on a cassette for enzyme-linked immunosorbent assay (Fig. 4c).

After process control units are determined, a structural part is required to integrate the functioning components into a working accessory that can be adapted to a mobile phone. Currently, 3D-printing technology is widely employed in laboratories for the quick and cost-effective manufacture of structural components. Previously, a smartphone-based blood analyzer was reported on by Zhu et al. using 3D-printing technology to develop a port and three removable components^70^ that could be assembled for white and red blood cell counting and hemoglobin measurement (Fig. 4d). In this example, the adaptation of the structural part to the smartphone uses the embedded mode, where the mobile phone is wrapped within a 3D-printed shell to fix the position of the external optical component to the mobile phone camera, and it is connected to the smartphone ports such as type-C to use computing power and electric power. The embedded mode has been the most common choice of researchers to develop mHealth platforms. However, the embedded mode requires customization of the structural part according to the overall dimensions of the mobile phone. Since different mobile phones often have different overall dimensions, it is difficult for researchers to design a universal part suitable for all mobile phones, let alone the possibility of establishing a unified production standard to adapt to modern society. Another approach to adapting the structural part to smartphones is patch mode, where the structural part is attached to the smartphone camera without connecting to the smartphone through various ports. The patch mode can be applied to various mobile phones, and it is suitable for developing miniaturized mHealth platforms with small volumes. For example, Comina et al. demonstrated a quantitative glucose meter that integrated finger pumps, unidirectional valves, calibration references, and focusing optics on a disposable unibody lab-on-a-chip device^71^. After the preparatory sequence had been activated, the device was placed on the front-facing camera of a mobile phone, and the default video acquisition application was run for quantitative analysis. After analysis, the device could be detached and disposed of, rendering the phone intact for its regular use. In comparison to the embedded mode, the patch mode allows universal adaptation of the developed mHealth platform to various mobile phone models. However, it is difficult for the patch mode to integrate relatively complex process control units, resulting in limited available detection means. As a common structural part, dark-box mode reserves a small hole and creates a dark environment for the smartphone camera to image fluorescent samples, which is adaptable to various sizes of smartphones and can integrate other functional units.

Despite this, there are still limitations in current approaches for adapting different smartphones. We expect modular smartphones to be a very promising platform for both future mHealth and mobile communication. Compared to the current mainstream all-in-one phones, modular smartphones can be customized with hardware configurations and accessories that can be changed at any time, making them more conducive to meeting the customized requirements of different users. For the POCT field, the heater for temperature control, electromagnet for bead fixation, and microfluidic chip for fluid manipulations, can be used as smartphone modules; these modules can be customized on the smartphones according to the testing scenario. Thus, the modular smartphone not only solves the difficulty in adapting mHealth accessories but also enables POCT instruments to be custom equipped according to testing methods.

## Machine intelligence for analyzing data

After the image data is collected by the integrated hardware, the collected image needs to be processed by the software algorithm. Therefore, the applicability of software algorithms directly determines the detection accuracy of mHealth platforms. In general, there are three types of software associated with mHealth platforms.

The first type uses mobile software development platforms such as Android studio to realize image acquisition, transmission and storage. Mainstream mobile phones use Android or iOS as the operating system. Researchers can use the officially recommended image storage or transmission library to call the corresponding interface through Java language to complete software development.

The second type mainly uses the lower computer, such as MCU, to accurately control the functioning components. Typically, assembly language, C language and other development languages are used to program functions for the underlying hardware using algorithms such as proportional integral derivative (PID), fuzzy and adaptive control, and reinforcement learning algorithms, which can accurately control the motor, heater and micropump integrated on the mHealth platform.

The above-mentioned two types of software do not involve complex image processing. The third type of software focuses on image processing and data analysis. They can be categorized into general, traditional machine learning, and deep learning algorithms, which will be discussed separately in the following sections.

### General image processing algorithms

A general image processing algorithm is the most direct and easy-to-use method to realize image preprocessing and image analysis. It does not need a lot of data for model training like machine learning algorithms. The general image processing algorithms used on mHealth platforms are similar to those commonly used on computers. However, due to the lower computing power of mobile phones, the mHealth platform prefers to use algorithms that do not require large computing power. Since the acquired image quality is often low on the mHealth platform due to its simple and cost-effective nature, it is necessary to use general algorithms for image preprocessing such as image enhancement, restoration, smooth denoising, graying and binarization to improve the image quality before analysis. Its main purpose is to enhance the authenticity of the image by eliminating irrelevant information and restoring useful information so that the image can be simplified to the greatest extent, facilitating the subsequent use of general image processing or other algorithms for feature extraction, image segmentation, matching and recognition.

#### Image enhancement

**Fig. 5:**
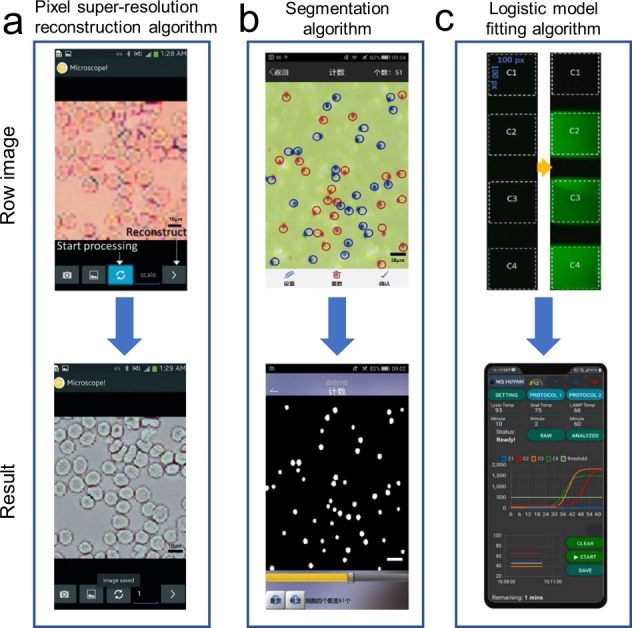
General image processing algorithms for mHealth platforms. **a** Pixel super-resolution reconstruction algorithm. The figure illustrates the use of a pixel super-resolution reconstruction algorithm to make a low-resolution image clearer (figure adapted with permission from Lee et al. ^59^). **b** Segmentation algorithm. The figure illustrates the use of a segmentation algorithm to determine ROI (figure adapted with permission from Zeng et al. ^73^). **c** The logistic model-fitting algorithm. The figure illustrates the use of the logistic model-fitting algorithm for real-time quantitative detection of nucleic acid (figure adapted with permission from Nguyen et al. ^75^).

As an image preprocessing method, the lens-free image reconstruction algorithm is a necessary step for lens-free imaging to obtain clear images. Current lens-free imaging has corresponding reconstruction algorithms, such as the pixel super-resolution method based on multi-angle illumination for shadow imaging (Fig. 5a) and holographic image reconstruction algorithm for lens-free holographic phase imaging. For example, Im et al. reported a generic approach to enable molecular diagnostics on a mHealth platform utilizing molecular-specific microbeads to generate unique diffraction patterns of blurry beads, which could be recorded and deconvoluted by digital processing^60^. To accurately detect bead-bound target cells, they developed a processing algorithm for image reconstruction that was based on the Rayleigh–Sommerfeld diffraction principle but extended to digitally retrieve both transmittance and phase shift of objects through an iterative optimization. As a result, cells and beads could be differentiated from transmittance and phase correlation respectively. Subsequently, cells labeled with microbeads could be automatically identified, and their individual bead counts were recorded.

#### Region of interest locating

After improving the image quality, to determine the specific region of interest (ROI) for analysis, location algorithms must be used to eliminate the irrelevant information of non-detection regions and locate the ROI. There are two commonly used location algorithms, namely, geometric transformation and image segmentation. Geometric transformation is to solve the problem of geometric distortion caused by imaging angle, perspective relationship, and even the lens itself. For example, Lopez-Ruiz et al. demonstrated a paper-based microfluidic colorimetric sensor for the simultaneous determination of pH and nitrite concentration in water samples. To locate the ROI in the microfluidic chip for colorimetric detection, geometric transformation needed to be used for preprocessing before extracting the color^72^. According to the reference coordinates, the rotation matrix and scale factor could be obtained through the location algorithm and used to correct the spatial distortion caused by different observation angles to determine the position of each sensing area. Then, the H (hue) and S (saturation) coordinates of the HSV color space are extracted and related to pH and nitrite concentration, respectively. As another location algorithm, image segmentation does not need to set reference coordinates in advance for locating ROI. It only needs to locate according to the difference in pixels between the detection area and irrelevant areas. For example, the counting algorithm for extracting object quantity often uses the image segmentation algorithm method, which is relatively simple and often used for cell and fluorescent marker counting, such as red and white blood cells^73^ (Fig. 5b).

#### Quantitative analysis

After image preprocessing, we can continue to use general image processing algorithms to extract the key information from the processed image for biomedical detection and analysis. For example, many studies directly use the color extraction function in OpenCV for colorimetric analysis. Since colorimetric detection is sensitive to external light, this processing method is difficult to obtain accurate results. Therefore, colorimetric detection with high accuracy often refers to the following process.

The first step is color gamut conversion. The photos taken directly by the camera are often stored in the form of RGB (red, green and blue) color space. RGB color space is a hardware-oriented color space, which is commonly used, but the images obtained in the natural environment are easily affected by natural lighting and shading, that is, they are sensitive to brightness. The three components of RGB color space are closely related to brightness. Moreover, the sensitivity of human eyes to these color components is different. In monochrome, human eyes are the least sensitive to red and the most sensitive to blue. Therefore, RGB color space is one with poor uniformity. If the color similarity is directly measured by Euclidean distance, the result will have a large deviation from human vision. For a certain color, it is difficult for our naked eyes to infer three more accurate RGB components to represent it. Therefore, RGB color space is suitable for the display system but not for image processing. In contrast, HSV (hue, saturation and value) color space is used more in image processing^74^. It is closer to people’s perception experience of color than RGB. It can intuitively express the hue, brightness and brightness of color and facilitate color comparison. Therefore, RGB color space needs to be converted to HSV color space before color comparison.

The second step is to eliminate light influence. After color gamut conversion, corresponding algorithms can be used to eliminate the influence of illumination, such as the gray world algorithm and color correction algorithm. The gray world algorithm assumes that the average value of R, G and B of an image with many color changes tend to the same gray value K. In the physical sense, the gray world algorithm assumes that the mean value of the average reflection of natural scenery to light is a fixed value in general, which is approximately gray. The gray world algorithm applies this assumption to the image to be processed, which can eliminate the influence of ambient light from the image and obtain the original scene image.

Finally, the use of the color extraction function can result in colorimetric detection with enhanced accuracy. Even though the above-mentioned method is adopted, there is still room for improvement. For example, the machine learning algorithm can be used to improve the accuracy of detection. Alternatively, an external light source with constant lighting conditions combined with a 3D-printed shell to isolate the influence of ambient light can greatly reduce the complexity of the detection algorithm, which can be used for colorimetric as well as fluorescence detection. Previously, Nguyen et al. reported on a mHealth platform that used CMOS to record the fluorescence change during real-time fluorescence quantitative LAMP to generate amplification curves^75^ (Fig. 5c). With the logistic model-fitting algorithm, the reverse transcription loop-mediated isothermal amplification (RT-LAMP) curve was fitted and was displayed on a smartphone for real-time quantitative analysis of nucleic acids.

### Traditional machine learning algorithms

For mHealth platforms, the acquired images are often affected by environmental factors due to the complexity of their operating environment. Since the robustness and anti-interference of general image processing algorithms are relatively weak, the analytical results of these algorithms are often different under different external light and acquisition environments. In this case, it is more suitable to use machine learning algorithms that can eliminate abnormal interference^76–78^.

Compared with the deep learning algorithm, the traditional machine learning algorithm requires less data, computing power, time and cost to train the model and is more interpretable. It is suitable for research fields with high sample collection costs and complex collection steps. Common traditional machine learning algorithms include support vector machine (SVM)^79^, bootstrap aggregation^80^, k-nearest neighbor (KNN)^81^ and decision tree^82^. Although these methods have different mathematical principles, they have some common processing steps, including (1) Establish the type of training examples; (2) Converge a training set; (3) Resolve the input feature illustration of the learned function/learned attribute; (4) Resolve the formation of the learned function and comparable machine learning algorithm; (5) Assimilate the design and execute the learning algorithm on the collected training set; (6) Evaluate the accuracy/correctness of the learned function. Among these, data acquisition, feature extraction and model choosing are key factors of the supervised machine learning algorithms^80^.

There are some principles to follow in selecting the appropriate model on mHealth platforms applying traditional machine learning or deep learning. First, the model can be selected according to the detection throughput of microfluidic chips, which determines the amount of collected data. Different models have different levels of complexity, which can be judged according to the Vapnik-Chervonenkis Dimension (VC-dim). The learning ability of the model is often positively correlated with the VC-dim, but when the data is insufficient for a model with a large VC Dimension, the effect is often poor and overfitting happens easily^83^. When the detection throughput is relatively low, the dataset is usually small. In this case, it is better to use statistical machine learning. This is because although deep learning models have a stronger learning capability compared to traditional models, they produce biases in estimating the actual data distribution with insufficient training sample and have a very high risk of overfitting, while many statistical machine learning models have a higher generalization capability under such conditions. In addition, few-shot learning, with much development in recent years, is also a solution to solve the problem of small datasets, which is very promising for low-throughput mHealth platforms. In few-shot learning, Siamese Networks are frequently employed, which use two identical artificial neural networks to build a coupled framework. In such a framework, the contrastive loss function is used to learn from a small dataset^84–86^. On the contrary, when the detection throughput is high and the dataset is huge, it is better to choose the deep learning model, which more easily achieves high accuracy with the huge dataset, while the accuracy of traditional models could plateau as the volume of data grows. Indeed, there are some candidate large-scale deep learning models with high effectiveness, such as Generative Pretrained Transformer 3 (GPT-3), which perform very well in many tasks using massive datasets and have very good prospects for application in high-throughput mHealth platforms based on cloud computing.

Second, the form of data also needs to be considered. Image, sequence, and graph data all have different processing methods. For image data, CNN is a commonly used model with translation and rotation invariance, which can accurately extract the features of images, and it is also a very common model used in mHealth platforms. In contrast, sequence- and graph-based models now have been used less in mHealth platforms but are very promising directions to explore in the future. For sequence data, RNN, LSTM, and transformer are very effective models. The transformer, which is particularly effective, can use a multi-head attention mechanism for parallel computing, has very good effects for sequence data analysis, and is expected to be applied to gene sequence analysis of mHealth. Graph data and related models, on the other hand, are areas that have shown greater development in recent years, such as Graph Attention Networks (GAT), which use attention mechanisms to effectively predict relationships between different nodes and can be used in mHealth to analyze interactions between different compounds and proteins, as well as realize combined diagnosis between different medical IoT edge devices to determine a user’s health status^87–89^.

Third, the model can be selected based on qualitative or quantitative analysis. For example, the classification algorithm is generally used for qualitative analysis, while the regression algorithm is used for quantitative analysis. Clustering and dimension reduction are also the basic tasks of machine learning. The model outputs corresponding to these tasks are very different, as are the algorithms used.

In the following sections, examples of POCT detection using machine learning algorithms will be discussed so that readers can more easily understand the usage of machine learning algorithms.

#### Algorithms for denoising

**Fig. 6:**
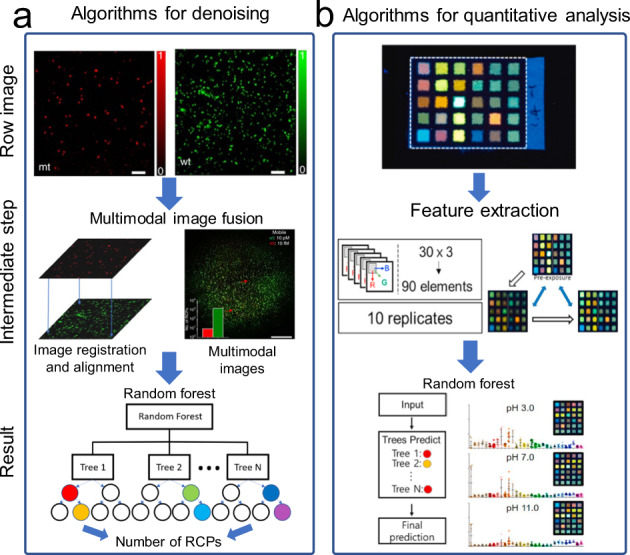
Traditional machine learning algorithms for mHealth platforms. **a** Algorithms for denoising. The figure illustrates the use of a random forest approach to differentiate real amplification signals from background noise after multimodal image fusion (figure adapted with permission from Kuhnemund et al. ^10^). **b** Algorithms for quantitative analysis. The figure illustrates the use of a random forest algorithm for colorimetric analysis and high-precision pH measurements (figure adapted with permission from Kim et al. ^96^).

For traditional machine learning algorithms, images are rarely processed directly, but the corresponding features are extracted through the general image processing algorithm and then classified or regressed to obtain the final analytical results. There is often a large amount of interference in real detection environments. Thus, it is necessary to use the machine learning algorithm to reduce noise before analysis and detection. For instance, signal-to-noise ratio (SNR) is often low in fluorescence images obtained by mHealth platforms due to strong background noise resulting from the use of weak light illumination and low-cost filters. Corresponding algorithms need to be used to reduce noise and improve SNR. Previously, Kuhnemund et al. demonstrated a cost-effective mobile-phone-based multimodal microscope for on-site molecular diagnostics^10^ (Fig. 6a). A machine learning-based algorithm was developed to process the acquired fluorescence images to count rolling circle amplification products. It utilized a random forest approach to differentiate real amplification signals from background noise, combining a bootstrap aggregation strategy and the randomness of the features to reduce overfitting.

In addition, Koydemir et al. presented a mHealth platform for the detection and quantification of *Giardia lamblia* cysts, one of the most common waterborne parasites^90^. A custom-developed machine learning algorithm was developed to count and differentiate cysts from other unwanted fluorescent micro-objects. The machine learning algorithm utilizes a bootstrap aggregating strategy to classify particles using 71 different features extracted for each cyst candidate. In subsequent works, they further studied the advantages and disadvantages of machine learning algorithms, including SVM, bootstrap aggregation and KNN. As a result, the bootstrap aggregation had the best performance, accuracy and fitting speed^91^. Surveilance et al. classified nematodes by applying a similar method^92^. In their following works, the recurrent neural network (RNN) algorithm was successfully used to differentiate live and dead nematodes^93^.

#### Algorithms for quantitative analysis

Machine learning can perform quantitative testing of samples directly. Solmaz et al. developed a smartphone application employing machine learning classifier algorithms for quantifying peroxide content on colorimetric test strips. The strip images were taken from five different Android-based smartphones under seven different illumination conditions to train binary (Least-Squares SVM) and multi-class (Random Forest) classifiers and to extract the learning model^94^. The extracted learning model was then embedded into a remote server that was accessed by a custom-designed Android app for testing purposes using a Cloud-hosted service. It turned out that the developed application was able to detect the color change in peroxide strips with over 90% success rate for primary colors with inter-phone repeatability under versatile illumination.

It is relatively straightforward to use HSV gamut data and machine learning algorithms to train classifiers for detection. However, with the increasing number of categories, the classification accuracy decreases significantly. Even if different machine learning models such as linear discriminant analysis (LDA) and Artificial Neural Networks (ANN) were employed^95^, it is still difficult to improve the multi-classification accuracy. This verifies a viewpoint in machine learning that the machine learning model only determines the lower limit of accuracy, while data and features extracted from data determine its upper limit. Therefore, we can only improve the source of data and feature extraction to achieve more detailed and accurate classification. Feature selection is often based on the specific characteristics of different applications. For example, Kim et al. developed a fluorescent array with a Kaleidolizine (KIz) system for pH classification^96^ (Fig. 6b). To develop pH-responsive fluorescent compounds for array composition, various anilines and phenols were introduced into the KIz core skeleton, allowing for the generation of 30 different colored fluorescent compounds. They confirmed that each fluorescent compound responded uniquely to pH changes. Thus, by spotting compounds on cellulose paper, they generated 5 × 6 fluorescent sensor arrays for pH classification. This small form factor of the sensor array enabled the smartphone camera to effectively capture fluorescence pattern changes of the array elements with respect to incubation with various pH buffers. Once images were captured, they were passed through several software components that extracted the color differences from the sensor array image. A random forest-based machine learning model was then used to classify the expected pH level of the buffer that the sensor was exposed to. They applied the developed method to the electronic nose for detecting organic volatiles, which could successfully distinguish 35 different volatile organic compounds^97^.

The above examples show that for microfluidic detection, researchers can not only select features according to the characteristics of samples but also combine feature signal amplification with the machine learning algorithm through a special microfluidic chip design to improve the detection accuracy. In many cases, the mHealth platform is a simple migration to traditional detection methods. Since people are usually the main operators who analyze test data, the detection principle and generated test data need to be intuitive and easy to understand. Traditional machine learning can produce analysis results from more complex data. However, it relies on the manual extraction of data features. In contrast, the deep learning algorithm can automatically extract data features, which opens up a new avenue for developing mHealth platforms.

### Deep learning algorithms

With the increasing amount of data, the recognition accuracy of the traditional machine learning algorithm will reach a plateau, while deep learning algorithms can start to give full play to their ability^98^. In recent years, with the rapid development of information technology, the progress of sensors has led to an increase in available data, and the progress of electronic technology has led to advances in computing power. Both facilitate the development of deep learning algorithms, especially the Convolutional Neural Network (CNN) algorithm applied in the field of biomedical imaging^99^.

Classical CNN algorithm includes the convolutional, pooling, and fully connected layers and the activation function. The layer is the core component of the ANN, which consists of fundamental neurons. Compared with classical ANN, the layers of CNN have their own special structure and function. The convolutional layer mirrors the structure of the human visual cortex, which can extract features of images. The pooling layer is used to aggregate features extracted by the convolutional layer to reduce the computational burden. After feature extraction, the fully connected layer will classify the data into various classes. The activation function is to give neural network nonlinear expression ability so that it can better fit the results. Compared with traditional machine learning, which requires professionals to manually extract image features, CNN can realize automated extraction of image features^100–106^.

By improving CNN architectures, including MobileNet, U-Net, Inception, Xception and Residual Network (ResNet), CNN can be used for various tasks, such as image enhancement, segmentation and classification, and regression detection. By realizing image processing and analysis with multi-layer stacking, CNN can achieve classification and regression without feature extraction. Therefore, it is more suitable for bright field imaging and morphology analysis.

#### Algorithms for image enhancement and segmentation

**Fig. 7:**
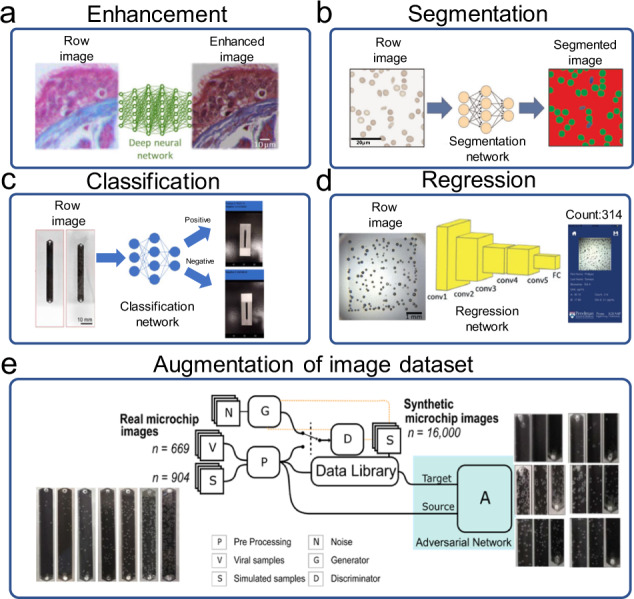
Deep learning algorithms for mHealth platforms. **a** Image enhancement. The figure shows the process of using deep learning to enhance low-quality images captured by mobile phones (figure adapted with permission from Rivenson et al. ^107^). **b** Image segmentation. The figure illustrates a deep learning workflow employing the U-net architecture for sickle cell analysis (figure adapted with permission from Haan et al. ^36^). **c** Image classification. The figure illustrates the detection of an on-chip bubble signal by using a CNN employing Inception v3 architecture (figure adapted with permission from Draz et al. ^109^). **d** Regression. The figure illustrates the regression CNN structure for counting bubbles (figure adapted with permission from Chen et al. ^110^). **e** Augmentation of the image dataset. The figure illustrates the structure of GAN for generating realistic synthetic microfluidic chip images to augment the image dataset (figure adapted with permission from Shokr et al. ^111^).

Due to the compact and low-cost hardware structure of the mHealth platform, the obtained images are often of low quality due to lens distortion such as spherical and color aberration. General image processing algorithms such as specific image degradation models can be used for image restoration and enhancement. However, these methods are effective in specific hardware settings and operation environments, which cannot be employed universally due to batch differences and low operation repeatability. To develop a universal algorithm to improve image quality on mHealth platforms, Rivenson et al. pioneered the use of the deep learning algorithm to correct lens distortion introduced by mobile-phone-based microscopes^107^ (Fig. 7a), facilitating the production of high-resolution, denoised, and color-corrected images, matching the performance of benchtop microscopes with high-end objective lenses. This work inspired researchers to explore deep learning algorithms for the development of mHealth platforms and biomedical applications.

Besides image enhancement, image segmentation can also be realized by the deep learning algorithm and combined with image enhancement through the complementary neural network. Haan et al. reported on a deep learning framework for the automated screening of sickle cells in blood smears using a smartphone-based microscope (Fig. 7b). Two distinct and complementary deep neural networks employing the U-net architecture were used for image analysis^36^. The first one enhanced and standardized the blood smear images to spatially and spectrally match the image quality of a laboratory-grade benchtop microscope. The second one acted on the output of the first image enhancement neural network and was used to perform the semantic segmentation between healthy and sickle cells within a blood smear. The segmented images were then used for the diagnosis of sickle cell disease. Blood samples of 96 patients with 32 positive cases were successfully tested by the developed method, showing ~98% accuracy with an area-under-the-curve (AUC) of 0.998. Although general image processing algorithms can also be used for image segmentation, it often requires trained experts who have knowledge of both medicine and image processing to extract image features before compiling the algorithm. In contrast, CNN allows experts to mark the data and hand it over to programmers with limited knowledge of medicine or image processing. Moreover, when the amount of data is relatively large, deep learning algorithms are often more universal and accurate than general image processing algorithms.

#### Algorithms for image classification

Deep learning algorithms have also been widely used for image classification on mHealth platforms. Compared with general image processing algorithms and traditional machine learning algorithms, deep learning algorithms do not need manual feature extraction and are more suitable for classifying images with complex features that are difficult for manual description and extraction. Recently, Potluri et al. developed a mHealth platform for automated ovulation tests using the deep learning algorithm by detecting fern patterns in air-dried saliva on a microfluidic device. Typically, images acquired by mHealth platforms are sent to a server for processing by CNN, and the results will be sent back to the mobile client device for display, taking advantage of the high performance of the server and reducing the burden on the mobile device^108^. In this work, the MobileNet architecture was used for the neural network to classify salivary ferning images, which is a CNN architecture specially designed for devices with low computing power, such as smartphones. Different from the traditional neural network using standard 2D convolution, this network uses depth-wise separable convolution to construct a neural network with a lightweight depth, allowing CNN-based image analysis directly on mobile devices. This technique is called model compressing, which can decrease the requirements of computation by reducing the sizes of models. The model size is often reduced by four model compression techniques, that is, pruning, quantization, knowledge distillation, and low-rank factorization. Using this technique, they were able to detect ovulation with an accuracy of 99.5% when tested with 200 images of human saliva collected during the ovulating and non-ovulating phases of the menstrual cycle among six women. More recently, the same research group reported a nanoparticle-enabled smartphone system for rapid and sensitive virus detection (Fig. 7c). Viral particles were captured on a microfluidic chip and labeled with specifically designed platinum nanoprobes to induce gas bubble formation in the presence of hydrogen peroxide^109^. The formed bubbles were controlled to make distinct visual patterns, allowing simple and sensitive virus detection on an Android smartphone with a trained CNN algorithm and without using any optical hardware smartphone attachment. The CNN algorithm employed the Inception v3 architecture, which was transfer learned using Google’s TensorFlow framework, with images of microfluidic chips containing bubbles analogous to virus samples. As a result, tests with 134 virus-infected patient plasma/serum samples showed a detection sensitivity of 98.97% and specificity of 91.89%.

#### Algorithms for regression

Regression is another common task that can be accomplished using deep learning algorithms. For example, Chen et al. reported the use of a microbubbling assay for the quantification of protein biomarkers by deep learning (Fig. 7d). Target proteins were captured by the antibodies immobilized on paramagnetic microbeads and further labeled with platinum nanoparticles^110^. After the sandwich complexes were loaded on a microwell array via an external magnetic field, the formation of microbubbles in the presence of hydrogen peroxide could be visualized. Both localization and regression CNNs were used for image analysis, allowing successful identification of the boundaries of the microarray areas and microbubble counting in seconds. Using this method, post-prostatectomy surveillance of prostate-specific antigens could be achieved with a detection limit of 0.060 pg mL-1 and early pregnancy detection using βhCG could be achieved with a detection limit of 2.84 pg mL-1. Ballard et al. reported on a deep learning-based framework to design and quantify point-of-care sensors. A low-cost and rapid paper-based vertical flow assay was demonstrated for testing high-sensitivity C-Reactive Protein^19^. CNN was used to select optimal spots and infer analyte concentration from the multiplexed sensing channels, which greatly improved the quantification accuracy in comparison to a standard multi-variable regression.

#### Algorithms for augmentation of image datasets

Deep learning algorithms have shown high potential for image processing and analysis, revolutionizing the use of smartphones in mHealth diagnostics. However, the high variability in cellphone image data acquisition and the common need for large amounts of specialist-annotated images for deep learning model training may limit the application of smartphone-based diagnostics. For example, in the above-mentioned work of Shafiee’s research group, a lot of time and resources were spent on the preparation of microfluidic chips and virus detection to obtain 15,057 images for CNN training^111^. To overcome this issue, they further employed adversarial learning to augment the real image dataset by generating 16,000 realistic synthetic microfluidic chip images through style generative adversarial networks (GAN) (Fig. 7e). The performance of the system was evaluated by detecting five different virus targets using 179 patient samples. The generalizability of the system was demonstrated by rapid reconfiguration to detect SARS-CoV-2 antigens in nasal swab samples (*n* = 62) with 100% accuracy.

Besides GAN, another approach to reduce the requirement of data volume for diagnostics is unsupervised learning without data annotation, also known as comparative learning. Shafiee’s research group utilized a medical domain adaptive neural network in both semisupervised and unsupervised learning scenarios to effectively capitalize on the largely unlabeled medical datasets, employing Xception and Res-net architectures. Their work showed that adversarial learning could be used to develop high-performing networks trained on unannotated medical images of varying image quality, and that it could be used with unlabeled data from unseen domain-shifted datasets to adapt pretrained supervised networks to new distributions, even when data from the original distribution are not available. They successfully applied the system to low-quality images acquired from inexpensive mobile optical systems to train networks for the evaluation of human embryos, the quantification of human sperm morphology, and the diagnosis of malarial infections in the blood^13^.

## Applications

In mHealth platforms, small and low-cost imaging devices are difficult to achieve high-resolution and high-quality images with a large field of view. Therefore, it is necessary to select an appropriate imaging modality, hardware structure, and algorithm according to the application scenario and detection object. In this section, the applications of mHealth platforms will be discussed in terms of the detection objects, that is, molecules, viruses, cells, and parasites. Examples will also be documented as a guide for readers to develop their own mHealth platforms.

### Molecules

Biological molecules, such as nucleic acids, proteins and metabolites, have scales below the Abelian limit (200 nm). These molecules cannot be directly imaged on mHealth platforms. Generally, a detection strategy such as polymerase chain reaction (PCR), fluorescence in situ hybridization (FISH), and enzyme-linked immunosorbent assay (ELISA) is utilized to allow optical detection under the mobile microscope.

For nucleic acid detection, PCR and FISH are the two common methods that are employed on mHealth platforms. Fluorescence imaging is typically employed for result collection, and result analysis could be accomplished with a fluorescence detection or counting algorithm based on machine learning.

PCR has been the gold standard for disease diagnoses, such as influenza, HIV, and genetic diseases. With the rapid advances in nucleic acid amplification technologies, isothermal amplification has drawn the increasing attention of researchers. Loop-mediated isothermal amplification (LAMP) has been a particularly promising alternative to PCR for nucleic acid detection on mHealth platforms. LAMP allows DNA amplification at 65 °C and the resulting amplificants can be detected via means including the naked eye, fluorescence, turbidity, colorimetry, and electrochemistry. Thanks to its tolerance of crude samples and critical reaction settings, extraction of pathogenic genes or sample pretreatment can sometimes be bypassed for direct LAMP reactions. Furthermore, LAMP offers a higher DNA synthesis rate and a milder reaction condition than PCR, which is ideal for rapid point-of-care detections with minimal setups. Hu et al. recently demonstrated a smartphone-based droplet digital LAMP device that integrated rapid nucleic acid extraction and digital LAMP for highly sensitive nucleic acid detection within 60 min^112^. A portable microdroplet fluorescence detection device was developed based on smartphone imaging. Quantification of low-abundance cfDNA and detection of mutations were successfully demonstrated.

FISH uses nucleic acid probes modified with chromogenic or fluorescent dyes to detect specific sequences on fixed histological specimens. The presence of the target nucleic acid sequence can be detected and quantified by a microscope, and multiple analyses can be performed based on the number of reporter molecules and microscope filters. Kühnemund et al. demonstrated a mHealth platform that permitted on-site molecular diagnostics with a cost-effective mobile-phone-based multimodal microscope, which allowed next-generation DNA sequencing reactions and in situ point mutation detection assays in preserved tumor samples to be imaged and analyzed^10^.

**Fig. 8:**
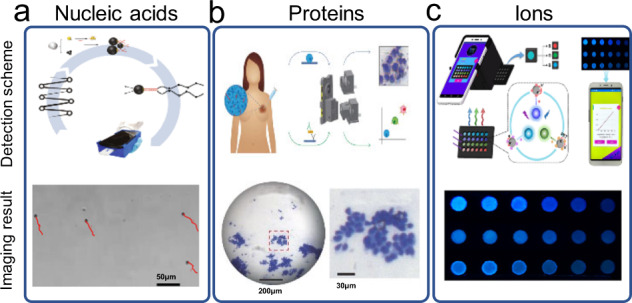
Molecular diagnosis on mHealth platforms. **a** Nucleic acids detection. The figure illustrates the detection of HIV-1 nucleic acids using a cellphone system integrating cellphone-based optical sensing, loop-mediated isothermal amplification, and micromotor motion (figure adapted with permission from Draz et al. ^11^). **b** Protein detection. The figure illustrates the workflow for breast cancer diagnosis using a mobile pathology platform (figure adapted with permission from Joh et al. ^114^). **c** Ion detection. The figure illustrates the workflow for the detection of Hg^2+^, Pb^2+^, and Cu^2+^ in water samples (figure adapted with permission from Xiao et al. ^122^).

As an interesting alternative to fluorescence imaging, microbead motion-based methods have opened a new avenue for nucleic acid detection on mHealth platforms. Recently, Draz et al. demonstrated a mHealth platform integrating cellphone-based optical sensing, LAMP and metal nanoparticle motion for molecular detection of HIV-1^11^ (Fig. 8a). The presence of HIV-1 RNA in a sample resulted in the formation of large-sized amplicons that reduced the motion of metal nanoparticles. The motion change could be accurately measured using a cellphone system as the biomarker for target nucleic acid detection.

For detecting proteins such as antigens, ELISA is the common strategy for signal amplification. Previously, Barbosa et al. reported on a power-free portable smartphone system for ELISA-based colorimetric and fluorescence quantitative detection of prostate-specific antigen (PSA)^113^. The developed system allowed quantitation of PSA in the range of 0.9 to 60 ng/ml with <7 % precision in 13 min using enzymatic amplification and a chromogenic substrate. The lower limit of detection was further improved from 0.4 to 0.08 ng/ml in whole blood samples with the use of a fluorescence substrate. The binding reaction between antibody and antigen can be directly used for protein detection. For example, Joh et al. developed a mHealth platform that was comprised of a custom cellphone-based optical microscope and an immunodiagnostic chip built upon a non-fouling polymer brush-coating that could quantify the expression of protein biomarkers directly from crude cell lysates^114^ (Fig. 8b). As a result, the method could evaluate both the cellular morphology and molecular expression of clinically relevant biomarkers directly from fine-needle aspiration of breast tissue specimens within 1 h.

For detecting ions, colorimetry is a simple and effective method that allows optical detection via the mobile phone camera to quantify ion concentration^115–121^. Microfluidic paper-based analytical devices (μPADs) are a relatively simple and economical means for developing POCT devices. Recently, Xiao et al. reported a stand-alone smartphone-based portable reader installed with a custom-designed APP, which could accurately and reproducibly acquire fluorescence change from a paper-based microarray for simultaneous detection of Hg^2+^, Pb^2+^, and Cu^2+^ in water samples^122^ (Fig. 8c).

The quick-response (QR) code is a widely used recognition technology, which has recently been combined with μPADs for colorimetric detection due to its ability to quickly locate and recognize encoded information, improving detection speed and increasing detection tolerance. Recently, Katoh et al. integrated QR code recognition into μPADs with distance-based colorimetric signaling, resulting in a semiquantitative readout fully relying on straightforward barcode reader solutions^123^. A model assay in the form of colorimetric copper ion detection was demonstrated in the concentration range of 0.4–3.2 mM. Consistent results were achieved with a free barcode reader APP independent of the smartphone model and environmental light conditions.

Due to the excellent signal amplification methods described above, the above particles can be detected even if their sizes are below the Abelian limit. By the indirect detection method, small particles can interact with the detection light source to produce detection signals that can be easily detected under fluorescence imaging. However, because of the portable and low-cost optical components (e.g., light sources, filters) of the mHealth platform, the image SNR of the mHealth fluorescence imaging platform is often relatively low, so denoising algorithms, such as machine learning algorithms, are required to improve the detection accuracy. Owing to the high sensitivity and specificity of fluorescence imaging, analysis algorithms used after preprocessing are often straightforward, and simple and counting or fitting algorithms will be used to complete the final quantitative detection.

### Viruses

Most viral particles have scales below the Abelian limit, but some special viral particles, such as coronavirus, are larger than it. Theoretically, when the size of the object is above the Abelian limit, it can be directly imaged and analyzed by a microscopic system. However, due to the limitation of the smartphone camera lens, the image quality acquired by the mHealth platform often has problems such as aberration and small FOV, resulting in an imaging resolution of >1 µm. As a result, the virus cannot be directly imaged on mHealth platforms for detection.

One strategy for detecting viruses on mHealth platforms is to detect their specific nucleic acid after lysis utilizing approaches such as PCR and LAMP. Another strategy is to directly detect the viral particles, which can also be accomplished by using the microbead motion-based method with the aid of immunological reaction. For example, Draz et al. reported the detection of Zika virus through monitoring the catalytic-based motion of nanostructure under a mobile optical system^124^. The presence of Zika virus in a testing sample resulted in the accumulation of platinum-nanomotors on the surface of polystyrene beads via immunological reaction, causing their motion in hydroperoxide solution. As a result, the method could detect Zika virus in samples with virus concentrations as low as one particle/μL. Since the motion characteristics of microbeads are easy to identify, the motion trajectory of microbeads can be captured directly in the bright field and analyzed by the particle tracking algorithm. In addition, fluorescent microbeads can be used to capture motions in the dark.

### Cells

**Fig. 9:**
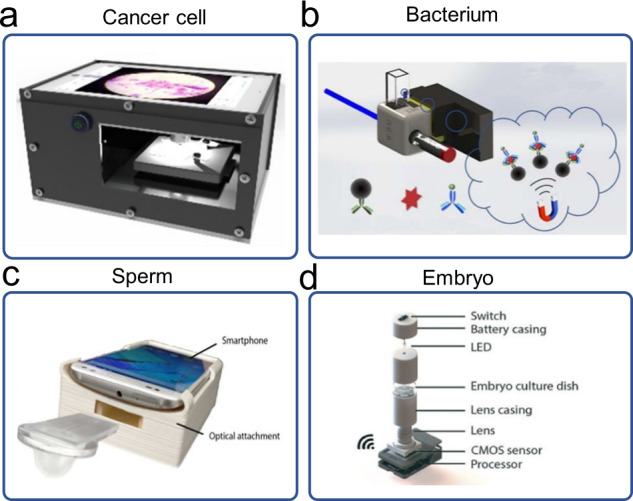
Detection of cells on mHealth platforms. **a** Cancer cell detection. The figure illustrates the design of a comparison study on the early detection of potentially malignant oral lesions by conventional cytology, tele-cytology, and ANN-based diagnoses (figure adapted with permission from Sunny et al. ^37^). **b** Bacterium detection. The figure illustrates a portable smartphone-based device with a sandwich immunosensor for *E. coli* O157:H7 detection (figure adapted with permission from Zeinhom et al. ^125^). **c** Sperm detection. The figure shows an image of the smartphone accessory and microfluidic chip for sperm detection (figure adapted with permission from Kanakasabapathy et al. ^126^). **d** Embryo assessment. The figure shows an exploded image of the stand-alone optical system and its various components. The system is wirelessly controlled using a smartphone to photograph embryos on a standard embryo culture dish. CMOS denotes complementary metal oxide semiconductor (figure adapted with permission from Kanakasabapathy et al. ^127^).

Cells and bacteria are generally larger than 1 µm. Thus, they can be detected on mHealth platforms by direct imaging. Sunny et al. demonstrated a tele-cytology system in combination with an ANN-based risk-stratification model for early detection of oral potentially malignant lesions (OPML)^37^ (Fig. 9a). Following automated scanning of cytology slides, acquired images were uploaded to a specialized web-based server for image preprocessing and ANN-based analysis. The integration of image processing and ANN-based risk-stratification model improved the detection sensitivity of malignant lesions and high-grade OPML. Zeinhom et al. demonstrate a compact and lightweight optical device attached to the existing camera module of a smartphone for detection of *Escherichia* *coli* O157:H7^125^ (Fig. 9b). Based on the classical sandwich ELISA design, rapid and specific detection of *E. coli* O157:H7 in foods was achieved within 2 h.

Semen analysis is the cornerstone of male infertility evaluation. Previously, Kanakasabapathy et al. developed an automated smartphone-based semen analyzer to quantify sperm concentration and motility for point-of-care male infertility screening (Fig. 9c). From a total of 350 clinical semen specimens, the developed mHealth platform could analyze an unwashed, unprocessed liquefied semen sample with <5 s mean processing time, providing a semen quality evaluation based on the World Health Organization guidelines with ~98% accuracy^126^. In the following work, the authors further evaluated the ability of the developed mHealth platform to provide information on Hyaluronan Binding Assay score, sperm viability, and sperm DNA fragmentation. Embryo assessment and selection is a critical step in an in vitro fertilization procedure. Kanakasabapathy et al. reported the development of two inexpensive and automated mHealth platforms that utilized deep learning algorithm for rapid, reliable, and accurate evaluations of embryo morphological qualities^127^ (Fig. 9d). Using a layered learning approach, they showed that the network models pretrained with high-quality embryo image data could be re-trained with data recorded on low-cost, portable optical systems for embryo assessment and classification when relatively low-resolution image data were used^128,129^.

Cell-scale objects are often larger than 1 µm, much greater than the Abelian limit, so they can be imaged directly with bright field imaging. However, because of the limitations of the smartphone microscope lens, the quality of microscopic images they take is not good enough and always has problems such as small FOV, aberration, and coma. Hardware structures with motors for field-by-field scanning are often used to solve the problem of small FOV, and deep learning methods for image enhancement are used to solve problems of aberration and coma. At the same time, the direct method using bright field imaging lacks high specificity like that of fluorescence imaging, so deep learning is required for image segmentation and classification to perform the final analysis of the image.

### Parasites

**Fig. 10:**
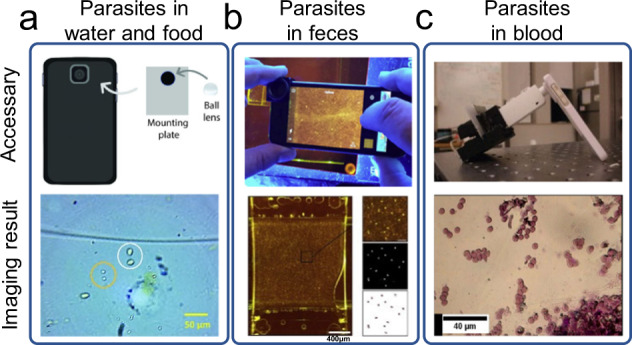
Detection of parasites on mHealth platforms. **a** Detection of *Giardia cyst* and *Cryptosporidium* (oo)cyst in food and water. The figure shows a representative image of (oo)cysts acquired by a smartphone microscope with a one mm ball lens and white LED light illumination (figure adapted with permission from Shrestha et al. ^198^). **b** Detection of parasite eggs in feces. The figure shows an example of a whole-field image of a sample in a McMaster chamber and image processing results during threshold segmentation (figure adapted with permission from Slusarewicz et al. ^199^). **c** Detection of malaria in blood. The figure shows an image of the mouse malaria strain blood smear without polarized light using a Leica microscope with a ×40 magnification objective (figure adapted with permission from Pirnstill et al. ^200^).

Parasite detection is closely related to food safety and personal health, which can also be realized by direct imaging detection on mHealth platforms^130,131^. The risk of host infection can be prevented by parasite detection. For example, *Giardia* eggs can be transmitted through drinking water or food, and the infected host will have symptoms such as diarrhea. Recently, Shrestha et al. reported a smartphone microscope method that could detect and quantify *Giardia* cysts and *Cryptosporidium* oocysts in food and water samples (Fig. 10a). The method was easy to implement, providing performance comparable to commercially available microscopic methods.

After the host is infected, parasites can be detected in its feces, urine, or blood according to the transmission mode of the parasites. Previously, Slusarewicz et al. demonstrated the use of a cellular smartphone as an inexpensive device to photograph parasite eggs that were labeled with a fluorescent chitin-binding protein in feces (Fig. 10b). By harnessing the computational power of the smartphone, parasite eggs could be counted through image analysis. As a result, the Strongyle egg counts generated by the smartphone system had a significant linear correlation with manual McMaster counts but with a lower coefficient of variation. Recently, Li et al. developed a cost-effective and automated system for counting parasites in fecal samples without special sample preparation or the need for a trained user. The system included an inexpensive, portable, robotic microscope that could scan over the size of an entire McMaster chamber and capture high-resolution bright field images without user intervention. The captured images were then automatically segmented and analyzed using a trained CNN to separate parasite eggs from background debris. Simple postprocessing of the CNN output yielded both egg species and counts.

Testing of blood samples is necessary for identifying blood parasites. For example, malaria is a life-threatening disease caused by parasites that usually infect subjects through mosquito bites. After infection, the parasite begins invading the host’s red blood and liver cells, modifying the biochemistry and structural properties of the cells. Pirnstill et al. reported a cost-effective, optical cellphone-based transmission polarized light microscope system for malaria diagnosis by imaging the malaria pigment known as hemozoin in blood samples (Fig. 10c). The developed system was comparable to larger benchtop polarized microscopy systems but at much lower cost and complexity. The detection of malaria in fixed and stained blood smears was successfully demonstrated.

## Conclusions and prospects

In this review, recent advances in the development of hardware and software of mHealth platforms and their applications were documented. As illustrated in Supplementary Fig. 1, in the early stages of development (before 2014), research on mHealth platforms was mainly focused on the supporting component of the imaging parts and modalities, which could be categorized into three types, that is, lens-free imaging, bright field lens-based imaging, and fluorescence imaging. Lens-free imaging often has a compact supporting component and requires image reconstruction for analysis. The resolution and FOV of lens-free imaging are directly related to the CMOS quality of the mobile phone, and it involves the removal of the smartphone camera lens, which may damage the integrity of the smartphone. Thus, currently, fewer and fewer mHealth platforms use lens-free imaging. Fluorescence imaging has the advantages of high specificity, large FOV, and wide-field imaging. However, the testing samples usually need to be pretreated with fluorescence staining before imaging. It is unfriendly to non-professional users to employ fluorescence imaging directly. In addition, since low-cost filters and LEDs are the major choices for fluorescence imaging on mHealth platforms for cost reduction, the resulting images often have a low signal-to-noise ratio (SNR). In contrast, bright field lens-based imaging is widely employed because microscopic modules can be easily adapted to mobile phone cameras for high-quality imaging, and the resulting images can be effectively analyzed by artificial intelligence algorithms such as CNN. However, when the resolution is very high, the FOV of bright field lens-based imaging becomes smaller. Thus, a trade-off between resolution and FOV is necessary according to application scenarios.

When imaging modalities became mature on mHealth platforms, researchers started to explore mHealth platforms for different application scenarios, such as blood cell and parasite detection, colorimetric and fluorescence detection of nucleic acids and proteins. At the same time, more hardware structures such as process control components and software algorithms such as colorimetric and dynamic video detection algorithms were developed. With rapid advances in smartphone performance and detection strategies, mHealth platforms have become simpler and more convenient, suitable for detecting a wide range of biological samples with improved sensitivity and accuracy.

Since 2018, research on mHealth platforms has further migrated to the development of software algorithms, such as deep learning algorithms for image enhancement and classification. The research group of Shafiee has shown many good examples, including male infertility tests (Fig. 9c), embryo assessments (Fig. 9d), and fern pattern-based ovulation tests. The combination of deep learning algorithms with mobile phone systems has opened a new avenue for mHealth platforms to further expand their application fields. However, machine learning algorithms, especially deep learning algorithms, need a large amount of data for model training. Thus, when choosing software algorithms for mHealth platforms, we should consider not only the advantages of the algorithm but also the time and resources that are required to generate the training set.

For future development, microfluidic-based detection methods hold high potential for sensing and evolving into mHealth platforms for mobile health monitoring. For example, localized surface plasmon resonance^132–147^ (LSPR) doesn’t need a prism or other optical coupling device to excite the sample and has a higher surface-to-volume ratio in comparison to traditional surface plasmon resonance. The integration of LSPR into mHealth platforms not only improves the portability of the detecting device but also makes the detection results more accurate and reliable by using deep learning algorithms. Liquid crystal (LC)-based sensors show colorful signals that can be readily interpreted by users without using expensive and bulky instrumentation under ambient light^148–152^. Therefore, they have been considered as simple and convenient methods that are suitable for routine analysis and on-site applications.

Recently, increasing attention of researchers is drawn to the integration of wearable devices with mHealth platforms^153–161^, where biological information is collected by wearable devices and then handed over to mHealth platforms for data analysis so as to realize real-time health monitoring. More and more mobile phone hardware resources such as near field communication and flashlight are applied to mHealth platforms to improve their portability^162^. In addition, many sensors embedded in smartphones such as gyroscopes and infrared and temperature sensors, also have a high potential to be implemented in mHealth platforms.

For machine intelligence, new models have emerged for image-based artificial intelligence analysis. For example, the transformer and pretrained models have been widely used in the computer vision area in the past two years. These newly developed models optimize the performance of deep neural networks from different aspects and greatly improve the accuracy of recognition. They hold high potential for applications in mHealth platforms.

When the high-performance model is relatively large, it usually requires a high computing power, making it difficult to deploy directly to mobile phones and thus a major barrier to mHealth development. Although algorithms deployed on cloud servers can analyze images uploaded from mobile phones, which is also known as cloud-based computing, it often suffers from higher latency and weaker privacy. Recently, fog- and edge-based computing (the way data is computed on gateways, routers or embedded devices) have attracted the attention of researchers with lower latency and higher data security, which can be integrated with cloud-based computing and have promising applications on mHealth platforms. In addition, although a lot of existing proven algorithms have been employed on mHealth platforms, few researchers developed new lightweight models specifically for mHealth platforms. Most researchers only program functions using canned algorithms in different applications. New lightweight models specifically for mHealth platforms may be the breakthroughs for existing challenges.

Above all, there is no doubt that mobile health monitoring is a very promising field. With the rapid development of the internet of things technology^163,164^, mHealth platforms based on smartphones have a wide range of application scenarios. It can be used for home nursing and health monitoring of family members, for use in the community medical station as the frontier of CDC, and field food safety detection. The collected data can be transmitted to the central server for analysis, which can be used by doctors for remote diagnosis of patients, scholars for pathological research, disease control officials for epidemic control, and improved theranostics^165–196^. We believe that soon, mHealth platforms will become more convenient and reliable with widespread applications.

## Supplementary information

Supplementary Information
