# Supplementary material for: Smartphone-based platforms implementing microfluidic detection with image-based artificial intelligence
Source: Nat Commun. 2023 Mar 11;14:1341. doi: 10.1038/s41467-023-36017-x (PMC10007670; doi:10.1038/s41467-023-36017-x)
Supplement: Supplementary file 1 — Supplementary Information [file 41467_2023_36017_MOESM1_ESM.pdf]

Supplementary Information

Smartphone-based platforms implementing microfluidic detection with image-based artificial intelligence

Bangfeng Wang[1,3], Yiwei Li[1,2,3], Mengfan Zhou[1], Yulong Han[2], Mingyu Zhang[1], Zhaolong Gao[1], Zetai Liu[1], Peng Chen[1], Wei Du[1], Xingcai Zhang[2]*, Xiaojun Feng[1]*, Bi-Feng Liu[1]*

[1]*The Key Laboratory for Biomedical Photonics of MOE at Wuhan National Laboratory for Optoelectronics - Hubei Bioinformatics & Molecular Imaging Key Laboratory, Systems Biology Theme, Department of Biomedical Engineering, College of Life Science and Technology, Huazhong University of Science and Technology, Wuhan 430074, China.*

[2]*School of Engineering and Applied Sciences, Harvard University, Cambridge, Massachusetts, 02138, USA.*

[3]These authors contributed equally: Bangfeng Wang, Yiwei Li

* Corresponding author emails:

xingcai0819@outlook.com (X.Z)

xfeng@mail.hust.edu.cn (X. F.)

bfliu@mail.hust.edu.cn (B. L.)

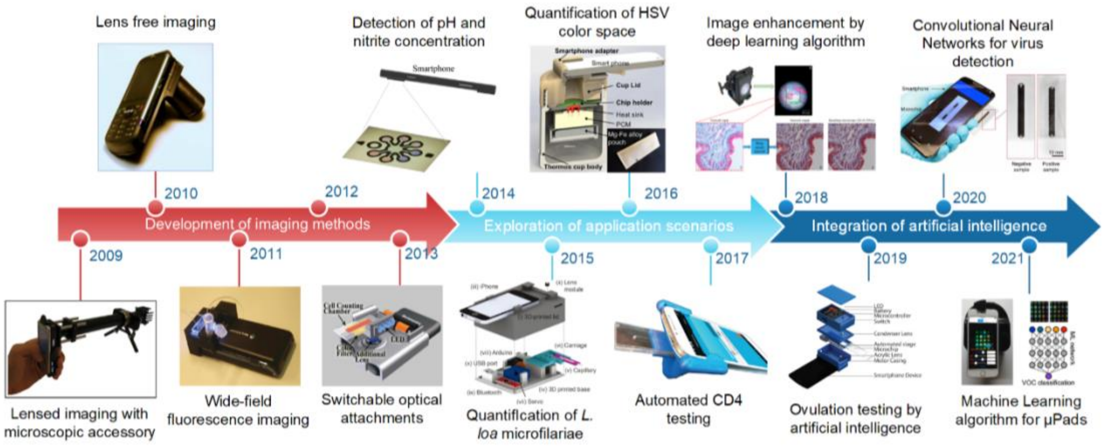

Supplementary Fig. 1. Timeline for the development of mHealth platforms with representative technological achievements and applications. HSV denotes Hue, Saturation, Value respectively, and µPADs denotes microfluidic paper-based analytical devices. (figures adapted with permission from ref. 1-12).

Imaging modalities	Advantages	Disadvantages	Resolution	FOV
Lens-free imaging [2,13–24]	Compact hardware structure suitable for mHealth platforms	Replacement of the smartphone camera; Requiring image reconstruction for clear results	0.5 ~ 2 µm	4.04 ~ 23.8 mm^2
Bright field lens-based imaging [1,5,25–32]	Direct morphological observation of tiny objects followed by analysis with algorithm such as CNN	Trade-off between resolution and FOV; Distortion around the edges of the images due to the curved nature of the spherical lens.	1.2 ~ 6.5 µm	0.0225 ~ 12.64 mm^2
Fluorescence imaging [3,12,27,33–39]	Relatively high specificity and large FOV	Pretreatment of samples; Unable to observe the morphological structure of tiny objects	1.7 ~ 20 µm	0.5 ~ 81 mm^2

Supplementary Table 1. Comparison of smartphone-based imaging modalities.

Machine intelligence	Algorithms	Tasks	Advantages	Disadvantages
General image processing algorithm	Counting algorithm; Lens-free image reconstruction algorithm; Colorimetric algorithm; Locating algorithm; Fluorescence intensity detection algorithm	Enhancement; Reconstruction; Denoising; Locating; Colorimetry	Simple and convenient; Low computing power requirements; Minimal data training requirements	Low robustness; Low accuracy under non-specific conditions
Traditional machine learning algorithm	Least-Squares SVM; Random Forest; Bootstrap aggregating	Classification; Denoising	Interpretable; Low data volume requirements	Manual feature extraction; Low accuracy in comparison to deep learning
Deep learning algorithm	CNN (MobileNet, U-Net, Inception, Xception, ResNet); GAN	Classification; Segmentation; Regression; Enhancement; Locating	Relatively high accuracy; Automatic feature extraction	Large data volume requirements; Large computing power requirements; Weak interpretability

Supplementary Table 2. Comparison of different types of algorithms in mHealth platforms.

Imaging modalities	Device components	Algorithms	Applications
Lens-free imaging [2,13–24]	CMOS [2,13,17]; LED [2,13,17]	Holographic reconstruction algorithm [2]; Pixel super-resolution algorithm [13]; Counting algorithm [17]	Cell counting [2,13]; Parasite detection [2,13]; Virus detection [17]
Bright field lens-based imaging [1,5,25–32]	External lens [1,3,9,26,40]; Motors [5,9,41]; LED [1,5,9,26,40]; Pump [42–45]; Diffuser ; MCU [5,9]	MobileNet [9]; U-Net [40]; Inception [10,46,47]; Xception [48,49]; ResNet [50]	Cell counting [7,51]; Parasite detection [52–56]; Ovulation detection [9]; Sickle-cell anemia diagnosis [40]
Fluorescence imaging [12,27,33–39]	External lens; Filters; LED; Heat sink; Laser module; Dichroic mirror	Bootstrap aggregating [57,58]; SVM [46,58,59]; KNN [59]; Random forest [59,60]; Counting algorithm [57]	Cell counting [12,33,40]; Parasite detection [34,57]; Virus detection [61]; Protein detection [27]

Supplementary Table 3. Linkages of imaging modalities, device components, algorithms, and applications.

References

1. Breslauer, D. N., Maamari, R. N., Switz, N. A., Lam, W. A. & Fletcher, D. A. Mobile phone based clinical microscopy for global health applications. *PLoS One* **4**, 1–7 (2009).
2. Tseng, D. *et al.* Lensfree microscopy on a cellphone. *Lab Chip* **10**, 1787–1792 (2010).
3. Zhu, H. *et al.* Cost-effective and rapid blood analysis on a cell-phone. *Lab Chip* **13**, 1282–1288 (2013).
4. Lopez-Ruiz, N. *et al.* Smartphone-based simultaneous pH and nitrite colorimetric determination for paper microfluidic devices. *Anal. Chem.* **86**, 9554–9562 (2014).
5. D'Ambrosio, M. V. *et al.* Point-of-care quantification of blood-borne filarial parasites with a mobile phone microscope. *Sci. Transl. Med.* **7**, 286re4 (2015).
6. Liao, S. C. *et al.* Smart cup: A minimally-instrumented, smartphone-based point-of-care molecular diagnostic device. *Sens. Actuators, B Chem.* **229**, 232–238 (2016).
7. Kanakasabapathy, M. K. *et al.* Rapid, label-free CD4 testing using a smartphone compatible device. *Lab Chip* **17**, 2910–2919 (2017).
8. Rivenson, Y. *et al.* Deep learning enhanced mobile-phone microscopy. *ACS Photonics* **5**, 2354–2364 (2018).
9. Potluri, V. *et al.* An inexpensive smartphone-based device for point-of-care ovulation testing. *Lab Chip* **19**, 59–67 (2019).
10. Draz, M. S. *et al.* Virus detection using nanoparticles and deep neural network-enabled smartphone system. *Sci. Adv.* **6**, 1–13 (2020).
11. Kim, H. *et al.* Kaleidoscopic fluorescent arrays for machine-learning-based point-of-care chemical sensing. *Sens. Actuators, B Chem.* **329**, 129248 (2021).
12. Zhu, H., Mavandadi, S., Coskun, A. F., Yaglidere, O. & Ozcan, A. Optofluidic fluorescent imaging cytometry on a cell phone. *Anal. Chem.* **83**, 6641–6647 (2011).

13. Lee, S. A. & Yang, C. A smartphone-based chip-scale microscope using ambient illumination. *Lab Chip* **14**, 3056–3063 (2014).
14. Mudanyali, O., Oztoprak, C., Tseng, D., Erlinger, A. & Ozcan, A. Detection of waterborne parasites using field-portable and cost-effective lensfree microscopy. *Lab Chip* **10**, 2419–2423 (2010).
15. Khademhosseinieh, B., Biener, G., Sencan, I. & Ozcan, A. Lensless on-chip color imaging using nano-structured surfaces and compressive decoding. *Opt. InfoBase Conf. Pap.* 2–3 (2011).
16. Oh, C., Isikman, S. O., Khademhosseinieh, B. & Ozcan, A. On-chip differential interference contrast microscopy using lensless digital holography. *Opt. Express* **18**, 4717 (2010).
17. Im, H. *et al.* Digital diffraction analysis enables low-cost molecular diagnostics on a smartphone. *Proc. Natl. Acad. Sci. U. S. A.* **112**, 5613–5618 (2015).
18. Nguyen, H. Q., Bui, H. K., Phan, V. M. & Seo, T. S. An internet of things-based point-of-care device for direct reverse-transcription-loop mediated isothermal amplification to identify SARS-CoV-2. *Biosens. Bioelectron.* **195**, 0956–5663 (2022).
19. Mudanyali, O. *et al.* Compact, light-weight and cost-effective microscope based on lensless incoherent holography for telemedicine applications. *Lab Chip* **10**, 1417–1428 (2010).
20. Zheng, G., Lee, S. A., Yang, S. & Yang, C. Sub-pixel resolving optofluidic microscope for on-chip cell imaging. *Lab Chip* **10**, 3125–3129 (2010).
21. Bishara, W. *et al.* Holographic pixel super-resolution in portable lensless on-chip microscopy using a fiber-optic array. *Lab Chip* **11**, 1276–1279 (2011).
22. Ozcan, A. & McLeod, E. Lensless Imaging and Sensing. *Annu. Rev. Biomed. Eng.* **18**, 77–102 (2016).
23. Isikman, S. O. *et al.* Lensfree computational microscopy tools for cell and tissue imaging at the point-of-care and in low-resource settings. *Anal. Cell. Pathol.* **35**, 229–247 (2012).

24. Stybayeva, G. *et al.* Lensfree holographic imaging of antibody microarrays for high-throughput detection of leukocyte numbers and function. *Anal. Chem.* **82**, 3736–3744 (2010).

25. Smith, Z. J. *et al.* Cell-phone-based platform for biomedical device development and education applications. *PLoS One* **6**, e17150 (2011).

26. Switz, N. A., D'Ambrosio, M. V. & Fletcher, D. A. Low-cost mobile phone microscopy with a reversed mobile phone camera lens. *PLoS One* **9**, e95330 (2014).

27. Joh, D. Y. *et al.* Cellphone enabled point-of-care assessment of breast tumor cytology and molecular HER2 expression from fine-needle aspirates. *npj Breast Cancer* **7**, 126 (2021).

28. Wicks, L. C. *et al.* EnLightenment: High resolution smartphone microscopy as an educational and public engagement platform. *Wellcome Open Res.* **2**, 1–18 (2017).

29. Sung, Y.-L., Jeang, J., Lee, C.-H. & Shih, W.-C. Fabricating optical lenses by inkjet printing and heat-assisted in situ curing of polydimethylsiloxane for smartphone microscopy . *J. Biomed. Opt.* **20**, 047005 (2015).

30. Rabha, D., Sarmah, A. & Nath, P. Design of a 3D printed smartphone microscopic system with enhanced imaging ability for biomedical applications. *J. Microsc.* **276**, 13–20 (2019).

31. Surveilance, M., Bornhorst, J., Nustede, E. J. & Fudickar, S. Mass surveilance of C. elegans—cmartphone-based DIY microscope and machine-learning-based approach for worm detection. *Sensors* **19**, 1468 (2019).

32. Hergemöller, T. & Laumann, D. Smartphone Magnification Attachment: Microscope or Magnifying Glass. *Phys. Teach.* **55**, 361–364 (2017).

33. Zhu, H., Yaglidere, O., Su, T. W., Tseng, D. & Ozcan, A. Cost-effective and compact wide-field fluorescent imaging on a cell-phone. *Lab Chip* **11**, 315–322 (2011).

34. Snow, J. W. *et al.* Rapid imaging, detection, and quantification of Nosema ceranae spores in honey bees using mobile phone-based fluorescence microscopy. *Lab Chip* **19**, 789–797 (2019).

35. Orth, A., Wilson, E. R., Thompson, J. G. & Gibson, B. C. A dual-mode mobile phone microscope using the onboard camera flash and ambient light. *Sci. Rep.* **8**, 1–8 (2018).

36. Kheireddine, S., Sudalaiyadum Perumal, A., Smith, Z. J., Nicolau, D. V. & Wachsmann-Hogiu, S. Dual-phone illumination-imaging system for high resolution and large field of view multi-modal microscopy. *Lab Chip* **19**, 825–836 (2019).

37. Rabha, D., Biswas, S., Chamuah, N., Mandal, M. & Nath, P. Wide-field multi-modal microscopic imaging using smartphone. *Opt. Lasers Eng.* **137**, 106343 (2021).

38. Rabha, D., Rather, M. A., Mandal, M. & Nath, P. Programmable illumination smartphone microscopy (PISM): A multimodal imaging platform for biomedical applications. *Opt. Lasers Eng.* **151**, 106931 (2022).

39. Rabha, D. *et al.* An affordable, handheld multimodal microscopic system with onboard cell morphology and counting features on a mobile device. *Analyst* **147**, 2859–2869 (2022).

40. de Haan, K. *et al.* Automated screening of sickle cells using a smartphone-based microscope and deep learning. *npj Digit. Med.* **3**, 76 (2020).

41. Park, Y. M., Kim, C. H., Lee, S. J. & Lee, M. K. Multifunctional hand-held sensor using electronic components embedded in smartphones for quick PCR screening. *Biosens. Bioelectron.* **141**, 111415 (2019).

42. Laksanasopin, T. *et al.* A smartphone dongle for diagnosis of infectious diseases at the point of care. *Sci. Transl. Med.* **7**, 273re1 (2015).

43. Comina, G., Suska, A. & Filippini, D. A 3D printed device for quantitative enzymatic detection using cell phones. *Anal. Methods* **8**, 6135–6142 (2016).

44. Suska, A. & Filippini, D. Autonomous lab-on-a-chip generic architecture for disposables with integrated actuation. *Sci. Rep.* **9**, 1–9 (2019).

45. Comina, G., Suska, A. & Filippini, D. Towards autonomous lab-on-a-chip devices for cell phone biosensing. *Biosens. Bioelectron.* **77**, 1153–1167 (2016).
46. Sunny, S. *et al.* A smart tele-cytology point-of-care platform for oral cancer screening. *PLoS One* **14**, 1–16 (2019).
47. Gunda, N. S. K., Gautam, S. H. & Mitra, S. K. Artificial Intelligence Based Mobile Application for Water Quality Monitoring. *J. Electrochem. Soc.* **166**, B3031–B3035 (2019).
48. Kanakasabapathy, M. K. *et al.* Development and evaluation of inexpensive automated deep learning-based imaging systems for embryology. *Lab Chip* **19**, 4139–4145 (2019).
49. Shokr, A. *et al.* Mobile health (mHealth) viral diagnostics enabled with adaptive adversarial learning. *ACS Nano* **15**, 665–673 (2020).
50. Kanakasabapathy, M. K. *et al.* Adaptive adversarial neural networks for the analysis of lossy and domain-shifted datasets of medical images. *Nat. Biomed. Eng.* **5**, 571–585 (2021).
51. Zeng, Y. *et al.* A low cost and portable smartphone microscopic device for cell counting. *Sens. Actuators, A Phys.* **274**, 57–63 (2018).
52. Bogoch, I. I. *et al.* Evaluation of a mobile phone-based microscope for screening of Schistosoma haematobium infection in rural Ghana. *Am. J. Trop. Med. Hyg.* **96**, 1468–1471 (2017).
53. Saeed, M. A. & Jabbar, A. “Smart diagnosis” of parasitic diseases by use of smartphones. *J Clin Microbiol* **56**, e01469-17 (2018).
54. Pirnstill, C. W. & Coté, G. L. Malaria diagnosis using a mobile phone polarized microscope. *Sci. Rep.* **5**, 1–13 (2015).
55. Shrestha, R. *et al.* A smartphone microscopic method for simultaneous detection of (Oo)cysts of cryptosporidium and giardia. *PLoS Negl. Trop. Dis.* **14**, 1–19 (2020).
56. Coulibaly, J. T. *et al.* Accuracy of mobile phone and handheld light microscopy for the diagnosis of schistosomiasis and intestinal protozoa infections in Côte d’Ivoire. *PLoS Negl. Trop. Dis.* **10**, 1–10 (2016).

57. Koydemir, H. C. *et al.* Rapid imaging, detection and quantification of Giardia lamblia cysts using mobile-phone based fluorescent microscopy and machine learning. *Lab Chip* **15**, 1284–1293 (2015).

58. Koydemir, H. C. *et al.* Comparison of supervised machine learning algorithms for waterborne pathogen detection using mobile phone fluorescence microscopy. *Nanophotonics* **6**, 731–741 (2017).

59. Solmaz, M. E. *et al.* Quantifying colorimetric tests using a smartphone app based on machine learning classifiers. *Sens. Actuators, B Chem.* **255**, 1967–1973 (2018).

60. Kühnemund, M. *et al.* Targeted DNA sequencing and in situ mutation analysis using mobile phone microscopy. *Nat. Commun.* **8**, 1–8 (2017).

61. Samacoits, A. *et al.* Machine learning-driven and smartphone-based fluorescence detection for CRISPR diagnostic of SARS-CoV-2. *ACS Omega* **6**, 2727–2733 (2021).
